# Impulsivity and inattention in hand tendon injuries: a case-control study revealing distinct profiles for work-related accidents

**DOI:** 10.1007/s00402-026-06354-9

**Published:** 2026-05-27

**Authors:** Ece Ağtaş Ertan, Gülseren Demir Karakılıç, Mehmet Batu Ertan

**Affiliations:** 1 Department of Psychiatry, Ankara Bilkent City Hospital, Ankara, Turkey; 2 Department of Physical Medicine and Rehabilitation, Bozok University, Yozgat, Turkey; 3 Department of Orthopedics and Traumatology, Atilim University, Ankara, Turkey

**Keywords:** Tendon injuries, Occupational injuries, Attention deficit disorder with hyperactivity, Impulsive behavior, Rehabilitation

## Abstract

**Introduction:**

The impact of attention deficit, impulsivity, and anger on various types of injuries is a subject of ongoing research. Hand tendon injuries are frequently encountered clinical conditions; however, their potential relationships with the aforementioned factors have not been previously investigated. This study aimed to examine the mechanisms of hand tendon injuries from certain psychiatric perspectives.

**Materials and methods:**

32 patients presenting to the physical therapy and rehabilitation outpatient clinic with hand tendon injuries and 32 healthy controls were evaluated. The assessment included sociodemographic data, causes of injury, Quick Disabilities of the Arm, Shoulder, and Hand (Q-DASH) and Visual Analog Scale (VAS) scores, as well as the physical and functional consequences of the injury. Patients and controls were assessed for attention deficit, impulsivity, and anger using the Adult ADHD Self-Report Scale (ASRS), Barratt Impulsivity Scale-11 (BIS-11), and State-Trait Anger Expression Inventory self-report scales. Statistical analyses of the results were performed.

**Results:**

Patients with tendon injuries were found to have significantly higher scores in ASRS attention and BIS-11 Motor impulsivity compared to the control group (*p* = 0.048, *p* = 0.040). Individuals with hand tendon injuries resulting from work accidents demonstrated significantly lower ASRS total, ASRS hyperactivity and impulsivity scores, and VAS rest scores compared to those with tendon injuries resulting from other causes (*p* = 0.049, *p* = 0.045, *p* = 0.038).

**Conclusions:**

Hand tendon injuries are associated with elevated impulsivity and inattention, suggesting that psychiatric screening could guide injury prevention and rehabilitation strategies. In contrast, the finding that work-related accidents are characterized by lower impulsivity indicates that prevention in this subgroup should prioritize environmental safety and occupational regulations over individual behavioral interventions.

**Trial registration number:**

NCT07126899.

## Introduction

Hand tendon injuries are one of the frequently encountered orthopedic injuries in clinical practice and can be challenging to treat [1]. While hand injuries account for 6.6–28.6% of all body injuries, they constitute 28% of musculoskeletal injuries. Hand injuries account for 20% of injuries in the emergency department [2]. This rate includes soft tissue injuries, fractures, and more complex injuries involving nerves, tendons, and vessels. Within this rate, tendon injuries are quite frequent; they are found in 54.8% of patients with minor lacerations and in 92.5% of patients with deep lacerations [3]. Therefore, the development of planned, effective, and simultaneously cost-effective rehabilitation approaches is of critical importance for the health system.

Due to their superficial location, tendon structures in the hand are areas susceptible to trauma. In the treatment of tendon injury, long-term and regular rehabilitation may be required in addition to surgical intervention. The primary aim of the rehabilitation process is to protect the repair site, ensure the prevention of complications such as adhesion, rupture, and joint contracture, and restore hand functionality. The occurrence of these complications both prolongs the treatment process and creates a significant economic burden in terms of workforce loss and the health system. It has been reported that approximately 42% of patients presenting to hand rehabilitation clinics have tendon injury-related conditions [4]. The injury itself and its consequences affect individuals not only in terms of labor but also socially and psychologically [5]. Beyond the initial surgical intervention and acute hospital care, tendon and nerve injuries of the hand necessitate extended sick leave, prolonged and resource-intensive rehabilitation, and continuous patient monitoring. In some instances, these injuries may even lead to the need for disability pensions, further exacerbating the substantial clinical and economic burden on both the individual and society [6, 7].

While traumatic tendon injuries in developed countries mostly occur as a result of industrial work accidents, in developing countries, including ours, they can originate from causes including violence-related injuries, suicide attempts, and punching glass after alcohol consumption, in addition to work accidents [8, 9]. Large-series studies conducted in our country have also reported that hand injuries constitute a significant portion of emergency admissions and lead to serious functional losses, particularly in the young adult workforce [3, 5]. Establishing the characteristics of these injuries, many of which occur through preventable mechanisms, is of great importance in terms of guiding preventive measures. Therefore, it is important to address tendon injuries not only in terms of treatment but also by identifying preventable risk factors.

Attention Deficit Hyperactivity Disorder (ADHD) is a clinical syndrome characterized by inattention, hyperactivity, and impulsivity. Although it is diagnosed more commonly in children, signs and symptoms can persist into adulthood [10, 11]. There are numerous studies investigating the relationship between the diagnosis and symptoms of ADHD and unintentional injuries [12–15]. Past research has shown that the probability of having an accident is higher in those diagnosed with ADHD, both in children and adults, compared to healthy controls [16, 17]. Epidemiological data also exist indicating that musculoskeletal injuries, in particular, are seen more frequently in individuals with an ADHD diagnosis.

In addition to core ADHD features, studies have shown that conditions frequently co-occurring with ADHD symptoms can also increase the risk of unintentional physical injury. For example, it has been stated that aggression and anger control problems can be significant determinants in the occurrence of traumatic injuries, and it has been reported that mental illnesses where anger control problems and impulsivity are prominent increase the risk of injury [18, 19]. It is suggested that temperament also exists as an important concept in the relationship between injuries and psychopathology. Impulsivity has also been identified as one of the significant factors in injury [20, 21].

There are studies investigating the relationship between various trauma-related injuries (fractures, burns, ear-nose-throat and eye-related traumatic injuries, etc.) and ADHD, conduct disorder, and various temperament traits in the literature [13, 15, 18, 21]. However, there is no research investigating the causes of hand tendon injuries and the relationship between these injuries and any of the domains of impulsivity, ADHD symptoms, or anger and anger expression styles. Demonstrating this relationship could guide clinicians in risk assessments for the prevention of tendon injuries and in the individualization of rehabilitation processes. Accordingly, the aim of our study is to examine the levels of ADHD symptoms, impulsivity, and trait anger in adults with and without hand tendon injuries, and to investigate their relationship with various health measures, injury mechanisms and functional losses. Based on previous research linking psychopathology to other forms of trauma, our primary hypothesis is that patients presenting to the physical therapy and rehabilitation outpatient clinic with hand tendon injuries will exhibit significantly higher levels of ADHD symptoms, impulsivity, and trait anger compared to healthy controls.

## Methods

This case-control study was conducted between October 2024 and June 2025. The study commenced after obtaining approval from the relevant local ethics committee in accordance with the Declaration of Helsinki (2024-GOKAEK-2411_2024.10.16_164). The study was registered with ClinicalTrials.gov (NCT07126899). 32 patients aged 18–65 years who applied to the Physical Medicine and Rehabilitation outpatient clinic of a tertiary training and research hospital due to upper extremity tendon injury, were literate, had no diagnosis of intellectual disability and/or dementia, and gave verbal and written consent to participate were included in the study. 32 healthy individuals who were similar in age and gender to the patient group, were literate, had no diagnosis of intellectual disability and/or dementia, and had no tendon injury were included as the control group. Exclusion criteria for the patient group included an unconfirmed history of tendon injury and tendon damage resulting from intentional self-harm. For both groups, exclusion criteria included diagnoses of schizophrenia, psychosis, and bipolar disorder, a history of past serious trauma or surgery affecting the upper extremity, rheumatologic disease, peripheral neuropathy, and the presence of severe upper extremity deformities.

Sociodemographic characteristics (age, gender, occupation, education, marital status) and general health information (smoking and alcohol use) of the patients and controls who participated in the study and provided consent were recorded. Participants’ general occupational status was recorded, although specific physical demands or inherent risk levels of their jobs were not formally quantified using a standardized scale. The Adult ADHD Self-Report Scale (ASRS), Barratt Impulsivity Scale-11 (BIS-11), and State-Trait Anger Expression Inventory were administered to both groups. These scales were chosen because they comprehensively evaluate both ADHD symptoms and ADHD-associated psychosocial parameters such as impulsivity and anger control, and because they have been frequently used in previous studies investigating the trauma-psychiatry relationship. In addition, information regarding the dominant side injury, mechanism of injury, treatment option, and presence of accompanying vessel-nerve-bone injuries was collected from the patient group. Additionally, the Quick Disabilities of the Arm, Shoulder and Hand (Quick DASH), which evaluates upper extremity pain and functionality, and the Visual Analog Scale (VAS), which evaluates pain severity, were administered.

### Scales used in the study

*Visual Analog Scale (VAS) for Pain Assessment*: Administered by having the patient mark the severity of pain and fatigue on a 10 cm horizontal line (0 = no pain, 10 = very severe pain).

*Quick Disabilities of the Arm, Shoulder and Hand (Quick DASH)*: This is a widely used, easily applicable scale allowing for international comparisons in evaluating functional limitations following tendon injuries. It consists of 11 multiple-choice questions used to obtain findings such as participation in daily living activities, independence, and pain, alongside physical measurements in upper extremity injuries. Responses in the survey range from 1 (no difficulty) to 5 (unable to do), with higher values indicating severe disability. The Turkish adaptation and validity-reliability study of Quick DASH was conducted by Doğan et al. [22].

*Barratt Impulsivity Scale-11 (BIS-11)*: A 30-item self-report scale used to evaluate impulsivity and its sub-dimensions (motor impulsivity, attentional impulsivity, and non-planning). Motor impulsivity is defined as acting without thinking; attentional impulsivity as making quick decisions; and non-planning as being focused on the present and not thinking about the future. The Turkish validity and reliability study of BIS-11 was conducted by Güleç et al. [23].

*Adult ADHD Self-Report Scale (ASRS v1.1)*: The WHO’s self-report rating scale for evaluating adult ADHD [24]. It consists of 18 items scored between 0 and 4 (0–4: never-very often), with 9 items evaluating inattention and 9 items evaluating impulsivity and hyperactivity. The validity and reliability study of the scale was conducted by Doğan et al. [25].

*State-Trait Anger Expression Inventory (STAXI)*: A 4-point Likert-type scale developed by Spielberger (1983). It consists of 10 items related to trait anger and 24 items related to anger expression style. The anger expression style scale consists of 3 sub-dimensions, each evaluated with 8 items; these sub-dimensions are anger control, anger-in (suppressed anger), and anger-out. The Turkish validity and reliability study was conducted by Özer et al. [26].

### Statistical analysis

Statistical analysis of the obtained data was performed using the SPSS 20 package program. Numerical data distribution was evaluated using the Shapiro-Wilk normality test; continuous data with normal distribution were summarized as mean ± standard deviation, and those without normal distribution as median (minimum-maximum); categorical data were summarized as frequencies and percentages. Chi-square test and Fisher’s exact test methods were applied in the analysis of categorical data. Inter-group comparison of normally distributed data was performed with the independent samples t-test, and inter-group comparison of non-normally distributed data was performed with the Mann-Whitney U test. Relationships between VAS, Quick DASH, ASRS, BIS-11, and STAXI were examined using Pearson or Spearman correlation coefficients according to the results of normal distribution tests. Power analysis was performed with G-power version 3.1.9.7 and calculated utilizing the total scores of the Wender Utah Rating Scale test from similar studies. According to these data, a calculation was made for a 0.05 margin of error and 80% power, and it was calculated that at least 32 patients in each group (patient and control groups), totaling at least 64 patients, should be included in the study. The confidence interval was accepted as 95% and *p* < 0.05 value as statistically significant.

## Results

There were no significant differences found between the participants in both groups included in the study in terms of age, gender, alcohol and smoking use, marital status, and education; the patient and control groups were similar in sociodemographic characteristics (Table 1).

**Table 1:** Sociodemographic characteristics of the patients and controls participating in the study

Age, (year) mean (SD)	Injury (*n* = 32)	Control (*n* = 32)	*p* value
	42.03 ± 15.89	43.81 ± 12.64	0.621
Sex, male (n) (%)	26 (81%)	21 (66%)	0.157
Education (n) (%) Primary Middle High University PhD	7 (22%) 5 (16%) 10 (31%) 9 (28%) 1 (3%)	4 (13%) 3 (9%) 8 (25%) 16 (50%) 1 (3%)	0.478
Marital Status, single (n) (%)	7 (22%)	7 (22%)	1.000
Smoking, yes (n) (%)	18 (56%)	14 (44%)	0.317
Alcohol, yes (n) (%)	0 (0%)	3 (9%)	0.238

SD: Standard Deviation

In the statistical analyses performed, it was observed that the mean scores of BIS total scores and cognitive, motor, and non-planning subscales were higher in patients with tendon injuries compared to healthy controls; this elevation in the BIS motor subscale was also statistically significant (*p* = 0.040). Similarly, in patients with tendon injuries, statistically significantly higher scores were observed in the ASRS Attention subscale, and higher scores were observed in the ASRS total and the hyperactivity-impulsivity subscales of this scale, although these were not statistically significant. Using a cut-off score of 40, more individuals in the tendon injury group (*n* = 6, 19%) were identified as high risk compared to the control group (*n* = 1, 3%), though this difference did not reach statistical significance (*p* = 0.104). In the STAXI, it was shown that trait anger, anger control, anger-out, and anger-in scores were similar in both groups, and no statistically significant difference was found between the groups (Table 2).

**Table 2:** Comparison of patients with traumatic hand tendon injury and healthy controls in terms of attention deficit and hyperactivity disorder, impulsivity, trait anger and anger expression styles

Barratt Total Score	Injury (*n* = 32)	Control (*n* = 32)	*p* value
	61.00 ± 12.90	55.88 ± 9.05	0.068
BIS-11 Cognitive Score	15.75 ± 4.30	13.91 ± 3.74	0.072
BIS-11 Motor Score	20.44 ± 5.42	18.06 ± 3.36	**0.040** *
BIS-11 Non-Planning Score	24.50 ± 5.13	23.91 ± 3.71	0.598
ASRS Total	26.72 ± 9.95	22.81 ± 8.28	0.124
ASRS Attention	13.38 ± 5.70	10.63 ± 4.51	**0.048** *
ASRS Hyperactivity Impulsivity	13.34 ± 5.36	12.19 ± 4.78	0.329
Continuous Anger	20.38 ± 6.46	19.09 ± 4.51	0.642
Controlled Anger	21.41 ± 5.91	21.88 ± 3.97	0.711
Expressed Anger	14.94 ± 4.44	15.03 ± 4.23	0.861
Repressed Anger	15.13 ± 3.42	15.81 ± 3.27	0.414

SD: Standard Deviation; ASRS: Adult ADHD Self-Report Scale; BIS-11: Barratt Impulsivity Scale

* p < 0.05 indicates statistical significance

When the injury causes of patients with traumatic hand tendon injuries were evaluated; it was observed that 9 of the patients were injured due to work accidents and 23 due to other causes (Table 3). It is noteworthy that 44% (*n* = 4) of those who had a work accident and 17% (*n* = 4) of those with tendon damage due to other causes sustained neurovascular damage, and fractures accompanying tendon damage were observed at a rate of 44% (*n* = 4) in those who had a work accident and 22% (*n* = 5) in others. No statistically significant difference was found between the two groups in terms of the specified variables. Additionally, when patients were grouped according to the tendon type (flexor vs. extensor), no statistically significant difference was observed between the groups in terms of ASRS, BIS-11, and anger scores (*p* > 0.05).

**Table 3:** Comparison of patients with hand tendon injuries due to work accidents and other causes in terms of injury characteristics and sociodemographic data

Age, (year) mean (SD)	Work (*n* = 9, %28)	Other causes (*n* = 23, %73)	*p* value
	35.78 ± 12.56	44.48 ± 16.62	0.167
Sex, male (n) (%)	8 (89%)	18 (78%)	0.648
Education (n) (%) Primary Middle High University PhD	1 (11%) 2 (22%) 3 (33%) 2 (22%) 1 (11%)	6 (26%) 3 (13%) 7 (30%) 7 (30%) 0 (0%)	0.442
Marital Status, single (n) (%)	3 (33%)	4 (17%)	0.370
Dominant Side, yes (n) (%)	3 (33%)	11 (48%)	0.694
Surgery, yes (n) (%)	9 (100%)	19 (83%)	0.303
Tendon (n) (%) Flexor Extensor Combined	5 (56%) 3 (33%) 1 (11%)	12 (52%) 10 (44%) 1 (4%)	0.722
Neurovascular Injury, yes (n) (%)	4 (44%)	4(17%)	0.176
Fracture, yes (n) (%)	4 (44%)	5(22%)	0.226

SD: Standard Deviation

When the results of the scale assessments of patients with traumatic hand tendon injuries due to work accidents and other causes were compared, it was observed that there was no statistically significant difference between the two groups in BIS-11 total scores and subscales, and trait anger and anger expression style scales (Table 4). The ASRS total and hyperactivity-impulsivity subscale scores of patients with hand tendon injuries due to work accidents were observed to be significantly lower than those with tendon injuries due to other causes (*p* = 0.049, *p* = 0.045). However, in pain and physical functionality assessments; it was determined that the VAS rest score was significantly higher in patients with tendon injuries due to causes other than work accidents (*p* = 0.038). It was observed that VAS Activity and VAS Sleep scores also had lower averages in those with work accidents, but this data was not found to be statistically significant. In the Q-DASH scale, which provides information on upper extremity pain and functionality level, it was observed that the total score averages of those with work accidents were lower compared to those with hand tendon injuries due to other causes, but it was seen that the results were not statistically significant (*p* > 0.05).

**Table 4:** Comparison of patients with hand tendon injuries due to work accidents and other causes in terms of attention deficit and hyperactivity disorder, impulsivity, trait anger and anger expression styles

	Work (*n* = 9, %28)	Other causes (*n* = 23, %73)	*p* value
BIS-11 Total Score	58.00 ± 14.39	62.17 ± 12.41	0.419
BIS-11 Cognitive Score	15.89 ± 6.29	15.70 ± 3.42	0.932
BIS-11 Motor Score	19.11 ± 4.96	20.96 ± 5.61	0.396
BIS-11 Non-Planning Score	23.00 ± 4.80	25.09 ± 5.24	0.309
ASRS 40 (n) (%)			
Low	9 (100%)	17 (74%)	0.150
High	0 (0%)	6 (26%)	
ASRS Total	21.22 ± 8.03	28.87 ± 9.95	**0.049** *
ASRS Attention	10.89 ± 4.34	14.35 ± 5.95	0.125
ASRS Hyperactivity Impulsivity	10.33 ± 4.24	14.52 ± 5.36	**0.045** *
Continuous Anger	19.11 ± 4.60	20.87 ± 7.08	0.773
Controlled Anger	21.11 ± 5.11	21.52 ± 6.30	0.863
Expressed Anger	14.44 ± 2.70	15.13 ± 5.00	0.902
Repressed Anger	14.44 ± 3.13	15.39 ± 3.55	0.490
VAS Rest	0.44 ± 1.33	2.78 ± 2.76	**0.038** *
VAS Activity	4.33 ± 1.80	5.87 ± 2.40	0.053
VAS Sleep	0.44 ± 1.33	1.13 ± 2.38	0.651
Quick-Dash	29.53 ± 15.15	38.62 ± 25.84	0.332

SD: Standard Deviation; ASRS: Adult ADHD Self-Report Scale; BIS-11: Barratt Impulsivity Scale; VAS: Visual Analog Scale; Quick-DASH: Quick Disabilities of the Arm, Shoulder and Hand.

* p < 0.05 indicates statistical significance

## Discussion

In our study, we evaluated impulsivity, attention deficit, trait anger, and anger expression styles in patients with hand tendon injuries, which are frequently encountered in the clinic and lead to physical, social, and psychological problems as well as workforce loss and in healthy controls. As a result of comparing the patient and control groups, which were sociodemographically similar, it was observed that the mean scores of attention deficit and impulsivity levels were higher in the group with tendon damage, and the scores of patients with tendon damage in the areas of attentional and motor impulsivity were found to be significantly higher than healthy controls. This finding suggests that tendon injuries may be associated not only with mechanical traumas but also with the cognitive and behavioral characteristics of individuals. Therefore, these results indicate that psychiatric evaluations should be taken into account in trauma prevention approaches. No statistically significant difference was observed between the tendon damage group and the control group in terms of trait anger and anger expression style.

ADHD is a neurodevelopmental disorder characterized by attention deficit, hyperactivity, and impulsivity. Although it was thought in the past to occur only in childhood, studies have shown that 40% of ADHD symptoms continue into adulthood and this rate is around 3–4% as one ages [10]. In a study conducted with a large sample in the USA, adult ADHD was found in the range of 1–6% [27]. In past research conducted mostly with child and adolescent patients; the relationship between ADHD diagnosis and injuries resulting from trauma has frequently been demonstrated. ADHD has been shown to be associated with various injuries (crush/sprain), open wounds and fractures of the head, neck, trunk, lower and upper extremities. In addition, severe injuries (skull, neck and trunk fractures, intracranial injuries without skull fracture, and nerve and spinal cord injuries) have been found to be more frequent in those with ADHD compared to those without [15]. It has also been shown that ADHD and impulsive behavior are higher in burn patients [28]. It has been suggested that ADHD may be associated with the severity of trauma, and it has been shown that the frequency of ADHD is significantly higher in high-energy traumas compared to low-energy traumas in patients presenting to the hospital with trauma [14]. In a population-based epidemiological study, ADHD was found to be associated with a roughly 2-fold increase in burns, poisonings, head injuries, and fractures [29]. As far as can be seen in the current literature, there is no research on tendon injuries and ADHD frequency or ADHD symptoms.

Although no participants had a prior ADHD diagnosis, the tendon injury group exhibited significantly higher inattention scores than controls. While it is vital to distinguish between a formal clinical diagnosis and self-reported subclinical symptoms, this subthreshold inattention may still predispose individuals to tendon injuries by impairing compliance with safety protocols, particularly in daily activities or occupations involving sharp instruments. Additionally, it was observed that the number of individuals determined to have a high probability for ADHD (ASRS > 40) was higher in the tendon injury group, but this elevation did not reach the level of statistical significance. Although ADHD evaluation is more difficult in adults, tendon injuries should be a warning in terms of screening these patients for attention deficit and referring patients thought to be at risk to psychiatry and following them up. The usual benefit of ADHD treatment is thought to be improving academic performance; however, preventing trauma and injuries is another aspect that should be considered. In a meta-analysis conducted by Man et al., it is stated that pharmacological treatment reduces injuries in ADHD by an average of 13%. It has been determined that the effect of drugs is more pronounced in adolescents and adults in this direction [30]. It is known that treatments such as CBT, mindfulness, and dialectic behavioral therapy are also effective in addition to pharmacological treatment in adults with an ADHD diagnosis [31]. Although the literature lacks specific studies on measures to reduce symptoms in individuals with subthreshold ADHD, it is suggested that this condition presents challenges, comorbidities, and risks similar to clinical ADHD, necessitating a comprehensive approach for symptomatic patients [32].

In addition to attention deficit and hyperactivity, it has been suggested that impulsivity, aggression, and anger control problems may also be significant determinants in the occurrence of traumatic injuries. Temperament traits, specifically impulsivity and inhibitory control, have been stated to be significant predictors in traumatic injuries. Impulsivity is defined as cognitive and behavioral tendencies involving difficulty in delaying or inhibiting a voluntary response, a short response threshold, a lack of deep thinking, and attention problems [33]. In our study, the motor dimension of impulsivity was shown to be significantly higher in individuals with tendon injury. Barratt et al. defined motor impulsivity as acting without thinking; additionally, they defined the attentional dimension of impulsivity as insufficiency in focusing on tasks, and non-planning as being more concerned with the present than the future [34]. According to another view, ‘motor impulsivity’ is associated with inhibiting potential responses [35]. The significantly higher motor impulsivity in the patient group can be interpreted as difficulty in inhibiting potential motor responses creating a predisposition to tendon damage in these individuals. Therefore, in patients with high motor impulsivity levels, it may be beneficial to integrate hand safety training and psychoeducation programs that increase awareness of risky behaviors into the rehabilitation process. Consequently, integrating psychoeducation elements that increase awareness of risky behaviors into rehabilitation programs may be beneficial.

It has been stated that conditions such as Oppositional Defiant Disorder (ODD) and Conduct Disorder (CD), which are known to manifest with difficulties in anger and behavioral control and impulsivity, and frequently co-occur with ADHD, are frequently observed in traumatic injuries [29, 36–38]. Furthermore, while some longitudinal studies provide evidence that aggression and conduct problems are prospective risk factors for future injuries, it has been suggested that hyperactivity is not supported by evidence as a prospective risk factor [18, 19].

Regarding the mechanisms of tendon injuries, which is the focus of our research, a study by Gökhan et al. on patients presenting to the emergency department showed that 53.8% of cases with hand and wrist injuries involving glass cuts occurred due to punching glass out of anger. Alcohol intoxication upon arrival and nerve and arterial injuries were also detected more frequently in these individuals [39]. In our study, based on the statements of patients presenting to the physical therapy and rehabilitation outpatient clinic, no injuries were identified with a mechanism involving punching glass due to anger. Since we could not evaluate conduct disorder and ODD due to the assessment of adult patients with hand tendon injuries in this study, when current trait anger and anger expression styles were evaluated using the self-report scales we administered, no significant difference was found between the tendon injury and healthy groups regarding anger-related variables. Since the study sample consisted of patients whose complaints persisted due to tendon injury, who attended follow-ups, and continued to seek treatment, they may exhibit different characteristics in terms of temperament and comorbidities compared to patients presenting to the emergency department. Furthermore, the similar anger expression styles and notably low alcohol consumption observed in both groups likely reflect the specific cultural and religious characteristics of the rural region where this study was conducted, limiting the generalizability of our findings to urban populations or different sociodemographic profiles.

In our study, evaluations regarding the causes and consequences of hand tendon injuries were also made, and specifically the similarities and differences between hand tendon injuries occurring due to work accidents and other causes were examined. Primarily, it was determined that 28.1% of hand tendon injuries in the population we investigated occurred due to work accidents. Although the differences between them did not reach the level of statistical significance, the high rates of neurovascular damage and fractures seen in those with work accidents is a noteworthy finding. However, the fact that work accidents can lead to more complex injuries emphasizes the importance of occupational safety measures. Although the stated results show that those with work accidents in our investigated sample are clinically and functionally similar to those injured by other causes in statistical evaluation, it can be interpreted that a study with a larger sample size regarding injury mechanisms and consequences in hand tendon injuries may be necessary. In particular, the observation of more complex injuries in work accidents suggests that the use of protective equipment, occupational safety trainings, and workplace inspections could play a critical role in preventing tendon injuries.

A striking finding in our study is that the ASRS total scores and hyperactivity and impulsivity scores of patients with hand tendon injuries due to work accidents were significantly lower than those with tendon injuries due to other causes. When patients were categorized by injury mechanism, the small sample size of the subgroup analysis limits statistical power, making these findings exploratory rather than definitive. Nevertheless, these preliminary results highlight that tendon injuries sustained in the workplace cannot be solely attributed to individual behavioral traits like inattention or impulsivity. This finding expands the recognized role of environmental factors and underscores the critical importance of primary prevention in occupational settings. To effectively avoid preventable accidents, focusing on strict environmental regulations, primary safety measures, and routine workplace inspections may be significantly more effective than addressing individual psychiatric characteristics.

However, in pain and physical functionality assessments, it was determined that the VAS rest score was significantly higher in patients with tendon injuries due to causes other than work accidents (*p* = 0.038). In the Q-DASH scale, which provides information on upper extremity pain and functionality level, along with pain scores during sleep and activity, it was observed that the total score averages of those with work accidents were lower compared to those with hand tendon injuries due to other causes, but it was seen that the results were not statistically significant (*p* > 0.05). In tendon injuries resulting from work accidents, closer follow-up of the patient may be in question as a requirement of employer responsibility and customary practices; this situation may have a positive effect on the outcomes of work accidents, but no previous research regarding this has been encountered.

In addition to clinical outcomes and follow-up requirements, a patient’s neurobehavioral characteristics are critical determinants of recovery success. Specifically, high motor impulsivity presents a unique challenge for hand rehabilitation, which requires strict adherence to immobilization protocols. Impulsive behaviors may predispose patients to premature splint removal or uncontrolled movements, increasing the risk of rupture. Therefore, identifying these traits early allows rehabilitation professionals to adopt closer monitoring strategies and personalized education to ensure compliance.

Our study has several limitations. First, since our study specifically included patients presenting to the physical therapy and rehabilitation outpatient clinic due to rehabilitation needs, patients presenting to the emergency department with minor injuries who did not require physical therapy may have been excluded. Nevertheless, this sampling method allowed us to target a specific population significantly affected by tendon injury, for whom preventive measures would be most impactful. Furthermore, the assessments of impulsivity, symptoms of attention deficit and hyperactivity (ADHD), trait anger, and anger expression styles in both the patient and control groups were based on self-report scales. Although self-report scales carry an inherent risk of recall bias, the instruments used in the study focus on stable traits rather than transient states, likely mitigating the influence of post-injury stress or rehabilitation. Nevertheless, this remains a potential limitation, and longitudinal studies are needed to clarify the causal direction of these risks definitively. Finally, given the exploratory nature of this study and the evaluation of multiple psychiatric subscales, our statistical approach was designed to identify potential initial trends rather than to definitively test multiple independent hypotheses. As such, strict multiple-testing conservative corrections were intentionally not applied to avoid obscuring early clinical signals. Consequently, our findings should be viewed as hypothesis-generating, providing a valuable foundation for future large-scale, confirmatory studies.

## Conclusion

Tendon injuries cause serious disability, loss of workforce, and economic burden. The injury itself and its consequences affect individuals not only in terms of workforce but also socially and psychologically [40]. Establishing the characteristics of these injuries, most of which occur through preventable mechanisms, is of great importance in terms of guiding preventive measures. Our study shows that attention deficit and impulsivity levels, which have been found to be associated with other injury types in past research, are also significantly higher in tendon injuries. Additionally, it serves as a preliminary evaluation that will guide future studies in terms of understanding the similarities and differences between the two groups where tendon injuries develop due to work accidents and other causes. These findings indicate that patients with hand tendon injuries should be evaluated not only orthopedically but also in terms of attention deficit and impulsivity. Although routinely implementing comprehensive psychiatric screening for every patient with a tendon injury is not cost-effective in standard clinical practice, remaining vigilant for suspected behavioral traits and referring high-risk individuals for psychiatric evaluation could facilitate smoother rehabilitation processes and ultimately reduce the likelihood of injury recurrence.

## Data Availability

All data related to this study is available upon a reasonable request to the corresponding author.
